# Prevalence and Associated Factors of Delayed Diagnosis of Type 2 Diabetes Mellitus in a Tertiary Hospital: A Retrospective Cohort Study

**DOI:** 10.3389/ijph.2022.1605039

**Published:** 2022-11-28

**Authors:** Kotchakorn Dulyapach, Pitchayanont Ngamchaliew, Polathep Vichitkunakorn, Phoomjai Sornsenee, Kittisakdi Choomalee

**Affiliations:** Department of Family and Preventive Medicine, Faculty of Medicine, Prince of Songkla University, Songkhla, Thailand

**Keywords:** prevalence, Thailand, type 2 diabetic mellitus, delayed diagnosis, tertiary hospital, Undiagnosed

## Abstract

**Objective:** To determine the prevalence and associated factors of delayed diagnosis of type 2 diabetes mellitus (DM) among outpatients in a tertiary hospital.

**Methods:** This retrospective cohort study was conducted among outpatients aged ≥35 years with twice fasting plasma glucose (FPG) levels ≥126 mg/dl between 1 January 2018, and 31 December 2020. The prevalence and pattern of delayed diagnosis of DM were defined using the Thai Clinical Practice Guideline (CPG) for Diabetes, 2017, and the American Diabetes Association (ADA) 2017. The cut-off time for FPG level confirmation of 3 months was used to evaluate delayed diagnoses and associated factors. Multiple logistic regression was used to identify variables associated with delayed diagnoses.

**Results:** Of 260 participants, 96.9% and 85.4% had delayed diagnoses as defined by the Thai CPG and the ADA, respectively. Factors significantly associated with delayed diagnosis were hypertension, non-cash insurance, and >10 years of physician experience.

**Conclusion:** Undiagnosed diabetes and diagnosis delay should be a concern in tertiary settings. Senior physicians should focus on patients with higher FPG levels, particularly those who have hypertension, and use non-cash insurance schemes.

## Introduction

Diabetes mellitus (DM) is a non-communicable disease and a major public health issue, and it is one of the top 10 causes of death worldwide [1]. A challenging aspect of type 2 DM is detecting the disease early enough to prevent progression to micro and macrovascular complications [2]. According to 2019 International Diabetes Federation (IDF) data, the global prevalence of diabetes was found to be 9.3%, with 463 million people diagnosed and 232 million people undiagnosed [1]. The Thai National Health Examination Survey VI reported that the prevalence of DM was 9.5% in 2020 and that 30.6% of diabetic patients were undiagnosed [3].

Delayed diagnosis of type 2 DM was defined when a lab diagnosis (i.e., hemoglobin A1c [HbA1c] ≥6.5%) of diabetes was conducted without a clinical diagnosis [4]. The overall global prevalence of delayed diagnosis was 23%–74% [5, 6]. A study from Atlanta Veterans Affairs Medical center in 2010 reported that, in patients with type 2 DM, the diagnosis was delayed for more than 3.7 years, and that physicians mentioned hyperglycemia without diagnosing or a follow-up plan in 60% of the patients, and 46% of the patients had hyperglycemia without the physicians mentioning the glucose value [7]. Factors associated with delayed diagnosis of type 2 DM can be divided into three categories: patient, physician, and healthcare system, defined as clinical inertia [7–9].

Delayed diagnosis represents a missed chance for early intervention to control hyperglycemia, prevent complications, and improve lifestyle [10]. A study from China reported that early diagnosis resulted in a significant reduction in all-cause mortality and cardiovascular mortality in patients diagnosed with diabetes and managed with lifestyle modifications. The age-adjusted all-cause death rate was four times higher and the cardiovascular death rate was seven times higher in patients without early intervention [11]. A study conducted in the U.S. in 2010 reported that screening for diabetes lowered the risk of myocardial infarction and diabetes-related microvascular complications (three to nine events prevented per 1,000 patients screened) and increased the Quality Adjusted Life Years to over 50 years [12].

As there is a high prevalence of delayed diagnosis worldwide, screening and early detection of type 2 DM and its timely management have become critical to prevent complications. As there are few studies on the delayed diagnosis of type 2 DM in Thailand, this study aimed to determine the prevalence of delayed diagnosis of type 2 DM among outpatients at a tertiary hospital in Southern Thailand, as defined by the Thai CPG for Diabetes, 2017 [13] and the Standard of Medical Care in Diabetes, American Diabetes Association (ADA) 2017 [14]. This study also aimed to identify the factors associated with delayed diagnosis. This could help with early diagnosis and management of type 2 DM and prevent micro and macrovascular complications and reduce cardiovascular mortality.

## Methods

### Study Design and Settings

This retrospective cohort study was conducted using data from patients who visited the outpatient department at Songklanagarind Hospital between 1 January 2018, and 31 December 2020, extracted from the Hospital Information System.

### Study Population, Sampling, and Sample Size Calculation

The participants in our study were outpatients aged ≥35 years with twice-measured fasting plasma glucose (FPG) levels ≥126 mg/dl. The exclusion criteria were as follows: patients with prior type 2 DM diagnosed before 1 January 2018 (as determined by ICD-10 codes [E11-E11.9], use of antidiabetic medications, or diagnosis text in the medical record data from the Hospital Information System [HIS]), those diagnosed at the first visit, those with gestational diabetes, and those with a history of oral corticosteroid use. Based on a study in the U.S. [4], the sample size for the study was calculated using a prevalence of 0.3 and a marginal error of 0.05. The target sample size was 323. Census sampling was used to recruit all participants who met the inclusion criteria.

### Data Collection

**FIGURE 1:**
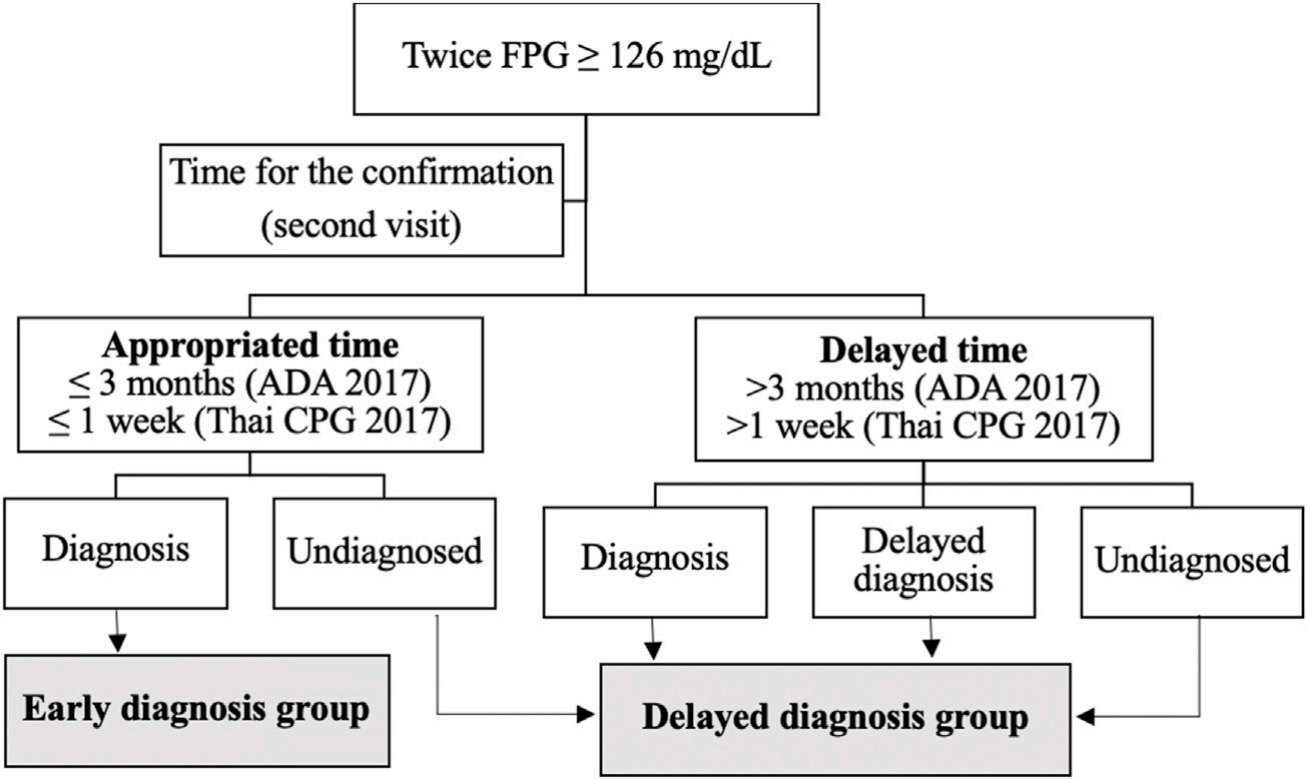
Identifying patients with early diagnosis and delayed diagnosis groups of type 2 diabetes mellitus (Songkhla, Thailand. 2021).

(1) The data were anonymized and extracted from the HIS by the Division of Digital Innovation and Data Analytics (DIDA), Faculty of Medicine, Prince of Songkla University. The data included the hospital numbers of participants aged ≥35 years with twice-measured FPG levels ≥126 mg/dl between 1 January 2018, and 31 December 2020.
(2) The data were collected and entered in Microsoft Excel version 2019. Each patient was assigned an identification (ID) number, and only those ID numbers were used to analyze the data sets. The patients were deidentified. The characteristic data of the patients including sex, age, comorbidities as determined by the ICD-10 codes (i.e., dyslipidemia, hypertension, cardiovascular disease, cerebrovascular disease, and chronic kidney disease), insurance schemes, body mass index (BMI) were classified into three levels as follows: <25 kg/m^2^, 25–29.9 kg/m^2^, and ≥30 kg/m^2^; the estimated glomerular filtration rate (eGFR) was classified as low-eGFR (<60 ml/min/1.73 m^2^) and normal eGFR (≥60 ml/min/1.73 m^2^), and low-density lipoprotein (LDL) cholesterol levels were retrieved during the first visit. Data on FPG levels were retrieved during the first and second visits. Data regarding the characteristics of physicians including years of work experience and the day of the appointment for patients’ second visits were collected. The healthcare system was characterized by the type of outpatient department into five clinics as follows: internal medicine clinics, medicine specialty clinics, general practice clinics, family medicine clinics, and other clinics.
(3) The second visit was defined as the day of the patient’s follow-up after the first visit when an FPG level ≥126 mg/dl was reported. Data on the characteristics of the physicians and healthcare system were collected during the second visit. We considered the possibility that some patients might have been treated by different physicians or across outpatient departments between their first and second visits. In addition, we collected data on the follow-up time of patients from the first visit to the second visit and divided the patients into two groups of early and delayed diagnoses, as described in Figure 1.

### Operational Definition

(1) Early diagnosis of type 2 DM was defined as FPG ≥126 mg/dl during the first visit and confirmation of FPG ≥126 mg/dl during the second visit, with diagnosis determined by ICD-10 codes (E11-E11.9), the use of antidiabetic medications (i.e., Metformin, Glipizide, Glibenclamide, Gliclazide, Pioglitazone, Sitagliptin phosphate, Linagliptin, Empagliflozin, Dapagliflozin, Liraglutide, Acarbose, Chlorpropamide, and all type of Insulin use), or diagnosis text in the medical record data. The study aimed to assess delayed diagnosis for benefits to improve the audit system monitoring indicators. According to the Thai CPG for Diabetes 2017, the appropriate time between the first and second visits for the confirmation of FPG levels must be between 1 day and 1 week [13]. However, the Standard of Medical Care in Diabetes, ADA 2017, suggested that, if patients had FPG levels near the margins of the diagnostic threshold, the physicians should repeat the test in 3 to 6 months [14]. In the present study, we determined the prevalence of early diagnosis based on clinical practice guidelines as follows: 1) the cut-off time for confirmation of FPG levels within 1 week based on the Thai CPG and 2) the cut-off for confirmation of FPG levels within 3 months based on the ADA, 2017.
(2) Delayed diagnosis of type 2 DM was defined as FPG levels ≥126 mg/dl in the first visit and confirmation of FPG levels ≥126 mg/dl during the second visit, undiagnosed by physicians for more than 1 week (Thai CPG for Diabetes, 2017) [13] or more than 3 months (Standard of Medical Care in Diabetes, ADA 2017) between first and second visits for the FPG confirmation [14]. In the present study, we determined the prevalence and pattern of delayed diagnosis based on both clinical practice guidelines and were divided into three types of delayed diagnoses (Figure 1). Subsequently, we evaluated early and delayed diagnosis groups and the factor associated with delayed diagnosis. We used the appropriate cut-off time for the second confirmation of FPG levels to within 3 months based on recommendations of ADA 2017, as this was an appropriate time for tertiary settings.

### Statistical Analysis

The data collected from the participants were entered into Microsoft Excel and analyzed using R software version 4.1.1. Data from descriptive statistics are presented as frequency and percentage. The characteristics of the early diagnosis and delayed diagnosis groups were compared using the *t*-test for continuous variables and the Chi-square or Fisher’s exact test for categorical variables. Multiple logistic regression was performed to identify the variables associated with delayed diagnosis outcome measures. Various variables when *p*-values were <0.1 were obtained in the univariate analysis, including, insurance schemes, normal eGFR status, patients with hypertension, and years of work experience of physicians. A manual backward stepwise refinement was performed for the final model. Adjusted odds ratios (AOR) and 95% confidence intervals (CI) were also calculated. When *p*-values were <0.05, the level of statistical significance was considered.

### Ethical Considerations

This study was approved by the Human Research Ethics Committee (HREC), Faculty of Medicine, Prince of Songkla University (REC.64-158-9-4). The DIDA, Faculty of Medicine, Prince of Songkla University fully computerized all medical records and reported them in confidence. Informed consent was waived because of retrospective data collection. The patient’s personal information is kept confidential.

## Results

All the medical records of the 973 participants with a twice-measured FPG level who met the inclusion criteria were reviewed. We excluded 713 participants who met the exclusion criteria (669 participants with prior type 2 DM diagnosed before January 1, 2018; 30 participants with a history of oral corticosteroid use; 13 participants diagnosed with type 2 DM at their first visit, and one participant with gestational diabetes). In total, 260 participants were enrolled to evaluate study outcomes.

### Prevalence and Pattern of Delayed Diagnosis

As shown in Table 1, the prevalence of delayed diagnosis of type 2 DM when defined using the Thai CPG for Diabetes, 2017, was 96.9%, of whom, 45.0% remained undiagnosed until 1 year later, and 51.9% had delayed confirmation time (more than 1 week between the first and second visits for the FPG confirmation) (28.8% of the participants had delayed confirmation time but received a diagnosis during the second visit, and 23.1% had delayed confirmation time along with delayed diagnosis during the second visit). Only 3.1% of the participants were diagnosed early with type 2 DM.

**TABLE 1:** Prevalence and patterns of diagnosis for type 2 diabetes mellitus (Songkhla, Thailand. 2021).

Patterns of diagnosis	Participant, n. (%)
**Diagnosis in 1 week (Thai clinical practice guideline for diabetes, 2017) [13]**	
Early diagnosis (1 week)	8 (3.1)
Delayed diagnosis (>1 week)	252 (96.9)
Delayed time for confirmation, but diagnosis at second visit	75 (28.8)
Delayed time for confirmation, and delayed diagnosis at second visit	60 (23.1)
Undiagnosed	117 (45.0)
**Diagnosis in 3 months (Standard of Medical Care in Diabetes, American Diabetes Association 2017) [14]**	
Early diagnosis (3 months)	38 (14.6)
Delayed Diagnosis (>3 months)	222 (85.4)
Delayed time for confirmation, but diagnosis at second visit	45 (17.3)
Delayed time for confirmation, and delayed diagnosis at second visit	60 (23.1)
Undiagnosed	117 (45.0)

When we altered the definition of delayed diagnosis of type 2 DM using the Standard of Medical Care in Diabetes, ADA 2017, the prevalence was 85.4%, of whom, 45.0% remained undiagnosed until 1 year later, and 40.4% had delayed confirmation time (more than 3 months between the first and second visits for the FPG confirmation) (17.3% of the participants had delayed confirmation time but received a diagnosis during the second visit, and 23.1% had delayed confirmation time along with delayed diagnosis during the second visit). Therefore, 14.6% of the participants were diagnosed early with type 2 DM, higher than the defined delayed diagnosis using Thai CPG for Diabetes 2017, as shown in Table 1. Subsequently, we evaluated our results based on the recommendations of the ADA 2017.

### Baseline Characteristics

The baseline characteristics of participants are presented in Table 2. Most of the participants were males (61.2%), and the mean age (±SD) was 63.2 (±11.9) years. Non-cash health insurance was used by 86.5% of the participants. The participants had comorbidities such as dyslipidemia (65.8%), hypertension (51.2%), and cardiovascular diseases (22.7%). The proportion of participants with FPG levels ranging between 126 mg/dl and 179 mg/dl was 93.5% on the first visit and 89.6% on the second visit with an overall median FPG level of 134 mg/dl during both visits. Participants in the early diagnosis group had a median FPG level significantly higher than that of participants in the delayed diagnosis group, in the first (143.5 vs. 133, *p*-value<0.001) and second (145.5 vs. 133, *p*-value<0.001) visits.

**TABLE 2:** Baseline characteristics and fasting plasma glucose levels of participants (Songkhla, Thailand. 2021).

Characteristic	Overall participants	Early diagnosis (≤3 months)	Delayed diagnosis (>3 months)	*p*-value
	(*n* = 260)	(*n* = 38)	(*n* = 222)	
**1. Participant characteristics**				
Male, n (%)	159	17 (10.7)	142 (89.3)	0.039^a^*
Mean age (SD), years	63.2 (11.9)	60.3 (13.6)	63.7 (11.5)	0.095^b^
Age (years), n (%)				0.021^a^*
<50	30	10 (33.3)	20 (66.7)	
50–59	71	8 (11.3)	63 (88.7)	
60–69	86	10 (11.6)	76 (88.4)	
≥70	73	10 (13.7)	63 (86.3)	
Insurance scheme, n (%)				0.001^a^*
Non-cash	225	26 (11.6)	199 (88.4)	
Cash	35	12 (34.3)	23 (65.7)	
Comorbid, n (%)				
Dyslipidemia	171	25 (14.6)	146 (85.4)	1^a^
Hypertension	133	12 (9.0)	121 (91.0)	0.015^a^*
Cardiovascular disease	59	4 (6.8)	55 (93.2)	0.084^a^
Cerebrovascular disease	13	3 (23.1)	10 (76.9)	0.413^c^
Chronic kidney disease	12	0 (0)	12 (100)	0.224^c^
BMI (kg/m^2^), n (%)				0.749^a^
<25	95	14 (14.7)	81 (85.3)	
25-29.9	107	14 (13.1)	93 (86.9)	
≥30	51	9 (17.6)	42 (82.4)	
eGFR ≥60 ml/min/1.73 m^2^, n (%)	207	36 (17.4)	171 (82.6)	0.022^a^*
LDL cholesterol ≥100 mmol/L, n (%)	154	21 (13.6)	133 (86.4)	0.877^a^
**2. Fasting plasma glucose**				
First visit				
Median FPG (IQR), mg/dL	134.5 (129,143)	143.5 (131,167.2)	133 (129,140.8)	<0.001^d^*
FPG (mg/dl), n. (%)				<0.001^c^*
126–179	243	30 (12.3)	213 (87.7)	
180–219	11	4 (36.4)	7 (63.6)	
220–299	3	3 (100)	0 (0)	
≥300	3	1 (33.3)	2 (66.7)	
Second visit				
Median FPG (IQR), mg/dL	134 (129,144.2)	145.5 (135.2,176.2)	133 (129,141)	<0.001^d^*
FPG (mg/dl), n. (%)				0.012^c^*
126–179	233	29 (12.4)	204 (87.6)	
180–219	12	3 (25)	9 (75)	
220–299	9	3 (33.3)	6 (66.7)	
≥300	6	3 (50)	3 (50)	

^a^ Chi-square test.

^b^ T-test.

^c^ Fisher’s exact test.

^d^ Rank sum test.

* Statistical significance (*p*-value<0.05).

Abbreviations: SD, standard deviation; LDL, low-density lipoprotein; BMI, body mass index; eGFR, estimated glomerular filtration rate; FPG, fasting plasma glucose; IQR, interquartile range.

The baseline characteristics of physicians and the healthcare system are presented in Table 3. Most physicians had less than 5 years of work experience (44.8% on the first visit and 47.8% on the second visit), and the most visited outpatient department was the internal medicine clinic (51.9% on the first visit and 53.8% on the second visit).

**TABLE 3:** Baseline characteristics of participants, physicians, and healthcare system (Songkhla, Thailand. 2021).

Characteristic	Physicians n	Overall participants	Early diagnosis (≤3 months)	Delayed diagnosis (>3 months)	*p*-value
		(*n* = 260)	(*n* = 38)	(*n* = 222)	
**Characteristics of physicians**					
Years of work experience					
First visit, n (%)					<0.001^a^ ^*^
<5	60	72	19 (26.4)	53 (73.6)	
5–10	20	28	6 (21.4)	22 (78.6)	
>10	54	160	13 (8.1)	147 (91.9)	
Second visit, n (%)					<0.001^a^ ^*^
<5	63	76	23 (30.3)	53 (69.7)	
5–10	14	18	2 (11.1)	16 (88.9)	
>10	55	166	13 (7.8)	153 (92.2)	
**Healthcare system characteristics**					
Outpatient department					
First visit, n (%)					<0.001^a^ ^*^
Internal medicine clinic	61	135	15 (11.1)	120 (88.9)	
Medicine specialty clinic	27	53	5 (9.4)	48 (90.6)	
General practice clinic	24	26	11 (42.3)	15 (57.7)	
Family medicine clinic	6	9	1 (11.1)	8 (88.9)	
Other clinic	28	37	6 (16.2)	31 (83.8)	
Second visit, n (%)					<0.001^a^*
Internal medicine clinic	61	140	17 (12.1)	123 (87.9)	
Medicine specialty clinic	25	51	6 (11.8)	45 (88.2)	
General practice clinic	24	24	11 (45.8)	13 (54.2)	
Family medicine clinic	8	10	0 (0)	10 (100)	
Other clinic	25	35	4 (11.4)	31 (88.6)	

^a^ Chi-square test.

* Statistical significance (*p*-value<0.05).

Our study revealed that third-fourth (63.1%) of the tertiary hospital physicians confirmed the second FPG between 1 month and 6 months and had a second FPG follow-up success rate of approximately 93%. While the follow-up success rate between 1 day and 1 week was 66.7%, which is presented in Table 4.

**TABLE 4:** Time of the physician appointment and patients who arrived for follow-up (Songkhla, Thailand. 2021).

Confirmation time	Physician’s appointment for second FPG visit n.	Patients appointed for second FPG visit n.	Patients arrived for the second FPG visit n.	Follow-up outcome rate^a^ %
1 day to 1 week	13	15	10	66.7
1 week to 1 month	17	20	18	90.0
1 month to 3 months	44	70	65	92.9
3 months to 6 months	62	105	98	93.3
6 months to 1 year	25	38	35	92.1
≥1 year	7	12	12	100

^a^ Follow-up outcome rate defined the percentage of participants who arrived on the day of their physician’s appointment for confirmation of second fasting plasma glucose.

Abbreviations: FPG, fasting plasma glucose.

### Factors Associated With Delayed Diagnosis in Type 2 Diabetes Mellitus

Based on the univariate model, factors such as gender (*p*-value = 0.039), age group (*p*-value = 0.021), insurance scheme (*p*-value = 0.021), presence of comorbidities including hypertension (*p*-value = 0.015), normal eGFR (*p*-value = 0.022), years of work experience of physicians (*p*-value<0.001), and outpatient department (*p*-value<0.001) were associated with delayed diagnosis of type 2 DM (Tables 2, 3). After adjusting for potential confounders (i.e., insurance scheme, normal eGFR, patient with hypertension, years of work experience of physicians), multiple logistic regression was used to identify the factors associated with delayed diagnosis. The factors significantly associated with the delayed diagnosis were hypertension (adjusted OR [AOR] 2.58; 95% CI 1.10–6.03), non-cash insurance (AOR 3.41; 95% CI 1.25–9.32), and >10 years of experience of physicians (AOR 6.70; 95% CI 2.89–15.54), as presented in Table 5.

**TABLE 5:** Multiple logistic regression for factors associated with delayed diagnosis of type 2 diabetes mellitus (Songkhla, Thailand. 2021).

Factor	Crude OR	Adjusted OR	*p*-value
	(95% CI)	(95% CI)	(Wald’s test)
**Patient-related**			
Hypertension (References: no)			
Yes	2.90 (1.36, 6.16)	2.58 (1.10, 6.03)	0.029^*^
eGFR (References: < 60 ml/min/1.73 m^2^)			
≥60 ml/min/1.73 m^2^	0.10 (0.01, 0.73)	0.14 (0.02, 1.10)	0.062
Insurance scheme (References: cash)			
Non-cash	3.73 (1.62, 8.57)	3.41 (1.25, 9.32)	0.017^*^
**Physician-related**			
Years of work experience (References: ≤ 5 years)			
5–10	3.38 (0.71, 16.02)	2.37 (0.47, 11.93)	0.297
>10	5.64 (2.62, 12.14)	6.70 (2.89, 15.54)	<0.001^*^

Multivariate analysis, using Wald’s test regression analysis, * Statistical significance (*p*-value<0.05).

Abbreviations: OR, odds ratio; CI, confidence interval; eGFR, estimated glomerular filtration rate.

## Discussion

### Statement of Principle Finding

The prevalence of delayed diagnosis of type 2 DM, when defined by the Thai CPG for Diabetes 2017, was 96.9% [13]. Over half (51.9%) of the participants had delayed diagnosis due to delayed confirmation time (between 1 day and 1 week). However, the Standard of Medical Care in Diabetes, ADA 2017, recommended that, if patients have FPG levels near the margins of the diagnostic threshold, the physicians should confirm FPG levels within three to 6 months [14]. Therefore, we altered the cut-off time based on the ADA 2017 for the confirmation of FPG levels to within 3 months to define the appropriate time for type 2 DM diagnosis, and the prevalence of delayed diagnosis decreased to 85.4%. In the present study, we determined the prevalence and pattern of diagnosis based on two clinical practice guidelines, namely Thai CPG 2017 and ADA 2017, wherein the cut-off time for confirmation of FPG levels was 1 week and 3 months, respectively. Subsequently, we evaluated the delayed diagnosis group and its associated factors based on the cut-off time for type 2 DM diagnosis per ADA 2017, as this might be an appropriate time in tertiary settings. It might be explained by the fact that early treatment to control blood glucose levels reduces the risk of vascular complication in type 2 diabetes and the time for confirmation for second FPG levels (only) within 3 months, which do not affect micro- and macrovascular complications [15]. While the confirmation time for second FPG levels is 1 week based on The CPG 2017, these might be affected by the overutilization of healthcare services, especially in a tertiary setting. A previous study reported that tertiary hospitals had a higher level of specialty care, as patients were referred from primary and secondary care hospitals in the nearby provinces [16]. The results of this study revealed that most of the physicians in the tertiary hospital (63.1%) gave appointments to their patients for the confirmation of FPG levels between one to 3 months (26.2%) and three to 6 months (36.9%) after the first visit. We suggest a three-month cut-off time for a second FPG level confirmation and consider it more appropriate in practice; this may serve as a tool in the clinical practice for the diagnosis of type 2 DM, especially in tertiary settings.

Moreover, almost half (45%) of the participants remained undiagnosed until 1 year later. This might be because of the physicians who did not specialize in diabetes from all outpatient departments in the tertiary hospital. In these cases, screening of FPG levels could be misevaluated or previous FPG results, such as pre-operative or annual laboratory findings, could be unreviewed [17]. Our prevalence of undiagnosed type 2 DM is consistent with previous evidence, Gopalan study report that 30.2% remained undiagnosed with type 2 diabetes 1 year later the first elevated index HbA1c [4]. Our undiagnosed DM was higher than the UAE study that reveal the prevalence of undiagnosed was 14.6% in patients without type 2 DM in a tertiary hospital [18]. Additionally, in the U.S. study report from 2011 to 2014, the percentage of diabetes cases that were undiagnosed was 10.9% [19]. Moreover, Samuels’ study report estimated that the median delay from the onset of type 2 DM to physician diagnosis was 2.4 years [15].

The prevalence of delayed diagnosis and undiagnosed type 2 DM varied across a large range depending on the diagnostic screening test, guidelines, healthcare system, and/or settings. According to the diagnostic screening test, some studies use the HbA1c levels to diagnose type 2 DM, resulting in an earlier detection in some cases [4, 16]. Moreover, some studies utilize the FPG levels for diagnostic screening, and 12%–40% of these patients revert to nondiabetic status after positive screening [20]. Hence, we monitored FPG levels twice to confirm the diagnosis. The settings may vary for prevalence based on the geographic location or healthcare system. A systematic analysis by Dessie et al. reports the average pooled prevalence of undiagnosed diabetes mellitus among adults in Africa at 3.85% and pooled prevalence varies based on geographic location in Eastern, Western, Northern, and Southern Africa [21]. The variety of healthcare service quality may be affected by undiagnosed or delayed diagnosis, such as the lack of access to healthcare or health insurance. A meta-analysis in the Eastern Mediterranean Region reported variable prevalence of undiagnosed diabetes, whereby countries with low Human Development Index (HDI) had the highest rate, while those with high HDI had the lowest prevalence [22].

This study’s results revealed the delayed diagnosis group had a significantly lower median FPG level than the early diagnosis group, during both visits. It might be explained in participants with slightly high FPG levels as the physicians may be more likely to miss the chance to make a diagnosis of diabetes. The U.S. study was supporting this notion, which showed that patients with lower index HbA1c values were less likely to be prescribed medication or have a verified diagnosis [4]. Moreover, most newly diagnosed type 2 DM patients with lower FPG levels were asymptomatic, making it difficult for physicians to diagnose and treat type 2 DM, and for the patients to adhere to the recommended lifestyle changes [23].

The following factors influence delayed diagnosis: patient, physician, and healthcare system, also referred to as clinical inertia [7–9]. Clinical inertia is the failure to initiate or intensify therapy according to evidence-based guidelines. Using logistic regression models, our study revealed that the factors significantly associated with the delayed diagnosis were comorbidities such as hypertension, use of non-cash insurance, and >10 years of physician experience. Participants with comorbidities such as hypertension might have a previous pattern of follow-up appointments, along with the associated date for the confirmation of FPG levels (which might cause a delay in confirmation). The delayed diagnosis might be not related specifically to the presence of hypertension but probably to the determinants of the overall cardiovascular comorbidities. This is consistent with the results of a study in Bosnia, wherein the patients with high blood pressure and comorbidities had a higher risk of clinical inertia because physicians paid more attention to healthy patients and those who were thought to be more disciplined [24]. Additionally, a meta-analysis by Aujoulat et al. showed that clinical inertia was more common in patients with comorbidities [25]. However, it is inconsistent with a study in Thailand that reported that the patients with hypertension were less likely to experience clinical inertia than the others [26]. Additionally, the findings of a study in the U.S., revealed that the presence of comorbidities precipitated the physician’s diagnosis [15]. Moreover, a study in Katoun showed that comorbidities (i.e., congestive heart failure, chronic kidney disease, and cardiovascular/cerebrovascular disease) resulted in a decreased likelihood of clinical inertia [27]. Delayed diagnosis in participants who used non-cash insurance was consistent with the result of the meta-analysis by Aujoulat et al., wherein clinical inertia was more common in patients from a low socioeconomic status because of the lack of access to healthcare or limited prescribed treatment [25]. The study from the U.S. reported that undiagnosed diabetes was more common in people lacking health insurance or access to healthcare [19]. Additionally, an Irish study reported that undiagnosed diabetes was independently associated with medical cost coverage. It might be explained by the effect of medical cost coverage on undiagnosed diabetes [28]. This finding suggests that physicians may respond better and check the FPG levels in patients who use cash payment than those using non-cash insurance schemes.

The physician factor for delayed diagnosis involved physicians with more than 10 years of experience. It might be explained by the fact that physicians with more experience in tertiary hospitals were specialists or sub-specialists, suggesting that they may have a long-term relationship with their patients and the management of type 2 diabetes was based on discretion and negotiation with patients, The study also reported that a reason related to the physician factor was delayed diagnosis, including the physician’s decision to not provide a diagnosis at a given time for patients as indicated [25]. In contrast, patients consulting the same physician over time were more likely to be diagnosed as they had more opportunities to talk about their symptoms and undergo diagnostic screening [6]. While physicians with less experience were residents who were a part of the educational system and supervised by a staff physician at an accredited tertiary hospital, they tended to follow clinical practice guidelines. This result is consistent with that of the study in the U.S., whereby physicians with less experience (<10 years of practice) provided appropriate diagnoses because they were updated with the guidelines and had recent publication knowledge [4]. Moreover, the study in Bosnia found that physicians not considering the clinical guidelines for treatment could cause delayed diagnosis [24]. Additionally, physicians with insufficient clinical knowledge provided inappropriate treatment caused by the misinterpretation of the guidelines or disagreement with guidelines that define HbA1c ≥6.5% as a diagnostic criterion but HbA1c <7% as the treatment goal [8]. Interestingly, the healthcare system was not significantly associated with delayed diagnosis in our study, supporting evidence that clinical inertia was not related to the physician’s work [25]. This result was inconsistent with the findings of a study in Croatia, whereby it was determined that physicians working in private practice were less likely to show clinical inertia because they might treat in primary care settings by a patient-centric technique rather than a concept-oriented approach [8].

### Strengths and Limitations

This study is one of the few to investigate delayed diagnosis of type 2 DM in Thailand and its association with patient, physician, and healthcare system factors. There were some limitations in our study. First, a small sample size was used because we collected data from 2018 to 2020 using the Thai CPG 2017 for diagnosis in a tertiary healthcare setting. However, our study used census sampling and the medical records of all the participants enrolled were reviewed. Second, our study defined type 2 DM by twice-measured FPG levels, which may not be as sensitive as the diagnosis by HbA1c, oral glucose tolerance test (OGTT), and random plasma glucose. However, we did not use HbA1c, random glucose values, and OGTT because these tests were not recommended for diabetes screening in the general population. Third, our retrospective cohort study that reviewed medical records might have some missing participant data such as physicians verbally providing a diagnosis and not recording it, or providing verbal advice for lifestyle modification. Therefore, these might have caused the overestimation of the prevalence of delayed diagnosis. In further research, a prospective study should be conducted to collect data and avoid this bias. Lastly, for the healthcare system characteristics, we did not collect data on the medical conditions leading to hospital access when participants attended the outpatient department, which might be variables associated with delayed diagnosis.

### Implications and Further Studies

The findings of this study showed that delayed diagnosis of DM is a major health concern in tertiary hospitals. These results will be useful to tertiary hospital physicians in the early detection and treatment of type 2 DM patients. This might also help to prevent macro and microvascular complications and reduce cardiovascular mortality. More research is required in a large, multicentric study. Moreover, findings of studies conducted in primary and secondary healthcare settings may reveal inconsistencies with the findings of this study. A study conducted during the COVID-19 pandemic could reveal a difference in the prevalence and pattern of delayed diagnosis because of the pandemic. A qualitative study involving focus groups is required to explore the reasons for delayed diagnosis, especially high experience levels among physicians.

### Conclusion

Delayed diagnosis of type 2 DM is a major health problem. Undiagnosed and delayed follow-up in a patient with hyperglycemia should be of concern in tertiary settings. To reduce delayed diagnosis, highly experienced physicians should be required to pay higher attention to patients with high FPG levels, especially those with hypertension and who use non-cash insurance schemes.
